# Acoustic Inspired Brain-to-Sentence Decoder for Logosyllabic Language

**DOI:** 10.34133/cbsystems.0257

**Published:** 2025-04-29

**Authors:** Chen Feng, Lu Cao, Di Wu, En Zhang, Ting Wang, Xiaowei Jiang, Jinbo Chen, Hui Wu, Siyu Lin, Qiming Hou, Junming Zhu, Jie Yang, Mohamad Sawan, Yue Zhang

**Affiliations:** ^1^ Department of Neurosurgery, The Second Affiliated Hospital of Zhejiang University School of Medicine, Hangzhou, Zhejiang Province, China.; ^2^ School of Engineering, Westlake University, Hangzhou, Zhejiang Province, China.; ^3^ Center of Excellence in Biomedical Research on Advanced Integrated-on-chips Neurotechnologies (CenBRAIN), School of Engineering, Westlake University, Hangzhou, China.; ^4^ State Key Laboratory of Cognitive Neuroscience and Learning, Beijing Normal University, Beijing, China.; ^5^ School of Foreign Languages, Tongji University, Shanghai, China.; ^6^ Center for Speech and Language Processing, Tongji University, Shanghai, China.; ^7^ Australian AI Institute, School of Computer Science, Faculty of Engineering and Information Technology, University of Technology Sydney, Sydney, Australia.; ^8^ Key Laboratory of Precise Treatment and Clinical Translational Research of Neurological Diseases of Zhejiang Province, Hangzhou, China.

## Abstract

Recent advances in brain–computer interfaces (BCIs) have demonstrated the potential to decode language from brain activity into sound or text, which has predominantly focused on alphabetic languages, such as English. However, logosyllabic languages, such as Mandarin Chinese, present marked challenges for establishing decoders that cover all characters, due to its unique syllable structures, extended character sets (e.g., over 50,000 characters for Mandarin Chinese), and complex mappings between characters and syllables, thus hindering practical applications. Here, we leverage the acoustic features of Mandarin Chinese syllables, constructing prediction models for syllable components (initials, tones, and finals), and decode speech-related stereoelectroencephalography (sEEG) signals into coherent Chinese sentences. The results demonstrate a high sentence-level offline decoding performance with a median character accuracy of 71.00% over the full spectrum of characters in the best participant. We also verified that incorporating acoustic-related features into the design of prediction models substantially enhances the accuracy of initials, tones, and finals. Moreover, our findings revealed that effective speech decoding also involves subcortical structures like the thalamus in addition to traditional language-related brain regions. Overall, we established a brain-to-sentence decoder for logosyllabic languages over full character set with a large intracranial electroencephalography dataset.

## Introduction

Language serves as the primary tool for human communication in both spoken and written forms. Previous demonstrations have proven the feasibility to decode language from the brain activity, particularly within the scope of alphabetic linguistic systems (e.g., English and Dutch), by establishing a mapping between language output and different activity-related brain signals, such as hand-writing [1], spelling [2–4], typing [5,6], and speech [7–14]. While such attempts have shown promise, decoding logosyllabic languages such as Mandarin Chinese and Thai, for which the speaking population is more than 1.3 billion [15], remains elusive. In contrast to the alphabetic languages, where words are constructed from combinations of limited size alphabets (e.g., 26 for English), Mandarin Chinese employ logographic characters to represent either entire words or single morphemes, which cannot be typed or spelled out directly. Chinese have an extensive inventory of over 50,000 logographic characters [16,17], each with its unique, complex glyph. For example, the English word “cat” is represented by a single character “
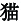
” in Mandarin, and “hot” is denoted as “
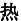
”. This complexity renders the decoding of handwriting via brain–computer interfaces (BCIs) exceedingly challenging for such languages.

Considering the fact that the pronunciation of Mandarin character is mostly detached from its glyphs, we seek a viable solution to bypass the curse of logographic systems via decoding the pronunciation of characters. Mandarin Chinese pronunciation is characterized by syllables, which are the combinations of initials (initial consonant of a syllable), finals (simple or compound vowel of a syllable), and tones (pitch variation at the syllable level) [18]. We propose a decoding system that achieves the conversion of speech-related brain signals into Chinese sentences by decoding the pronunciation of Mandarin syllables. This system comprises 3 acoustic inspired syllable element prediction models (for the initial, tone, and final, respectively), which explicitly captures the distinct acoustic–phonetic patterns during Mandarin articulation to enhance the prediction performance for syllable components. Given the presence of homophones in Mandarin Chinese [e.g., the combination of initial “t” ([t^h^]), final “a” ([a]), and a high-level tone (tone 1) can be mapped to both “
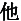
” (tā, [t^h^a], he) and “
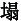
” (tā, [t^h^a], breakdown)], the surrounding context plays a pivotal role in identifying the correct character in a sentence when mapping pronunciation into characters. Our system employs a language model to resolve pronunciation to character ambiguities according to the semantic context and convert syllable sequence into a complete sentence with practical meanings.

**Fig. 1.**
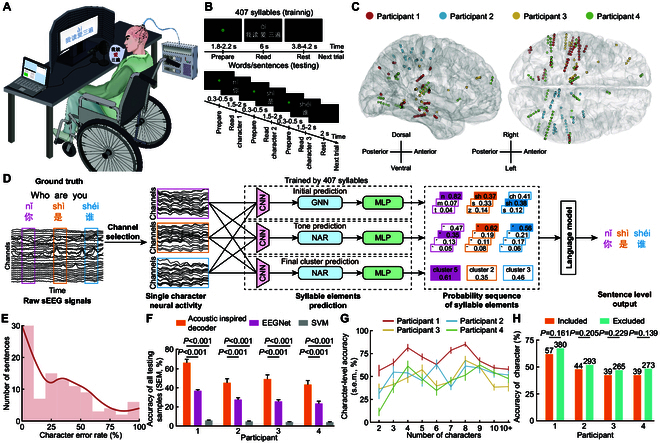
The pipeline and performance of the brain-to-sentence decoder designed for logosyllabic language decoding. (A) After the electrodes were implanted, participants took part in the study in the wheelchair, reading sentences given visual cues. Audio data were collected using a directional microphone, and neural signals were simultaneously recorded using a multi-channel electrophysiological recording system. (B) Two tasks designed for collecting training used data (upper panel, 407 syllables cover almost all Mandarin Chinese characters) and evaluating used data (lower panel, 100 sentences ranging from 2 to over 10 characters in length). (C) The distribution of selected decoding channels across participants is illustrated on the standard Montreal Neurological Institute (MNI) template brain. Each participant’s channels are denoted by distinct colors, with directional indicators provided for clarity. (D) A 3-phase decoder, including channel selection, syllable element prediction, and language model correction, is designed to output whole Mandarin Chinese sentences by decoding speech-related sEEG signals. CNN, convolutional neural networks; MLP, multilayer perceptron. (E) Distribution of decoding accuracy of 100 test samples (data from participant 1). Outlines are kernel density estimates of the distributions. (F) Decoding accuracy (mean ± SEM) of all evaluating samples compared with EEGNet and SVM model, which is the average of the proportion of correct characters in each sample. (G) Character-level accuracy (mean ± SEM) of sentences composed of different lengths. (H) The comparison of decoding accuracy of characters appeared in the training reading corpus (Included) and those absent (Excluded), with the number of correctly decoded characters displayed on the corresponding bar. The significance was calculated by bootstrapping.

Most previous studies on language decoding use electrocorticography (ECoG) electrodes [3,9,11,14,19–21] or Utah array electrodes [1,7,22] to acquire cortical signals. However, some studies show that the subcortical signals have potential to enhance the effectiveness of decoder [23,24]. Our study utilized stereoelectroencephalography (sEEG) to capture both cortical and subcortical neural signals simultaneously from 4 participants (2 males and 2 females, all young native Mandarin speakers) undergoing invasive monitoring for epilepsy (Fig. 1A). For training the decoder, participants were instructed to read 407 monosyllabic characters, which correspond to the 407 distinct Mandarin syllables (the upper panel in Fig. 1B). These 407 syllables are specially designed to contain all the initial and final pairs, so the pronunciation of almost all Mandarin Chinese characters is included. Trained using such data, the proposed BCI achieved the direct decoding of brain activity directly into text sentence consisting of any Chinese character, where the sentences are highly unconstrained and free from the limitations imposed by the training data. Particularly, we decoded text from brain recordings of participants reading 100 Mandarin sentences selected randomly varying in length from 2 to more than 10 characters (the lower panel of Fig. 1B).

Unlike existing work on character-level Chinese decoding, which covers only a small number of Chinese characters [13], our sentence-level decoding system can handle full sentence decoding over full set of Chinese characters, with a median character accuracy of 71.00%, and 30.00% of decoded sentences are completely accurate in the best participant, potentially enabling the reconstruction of free language expression in speech-impaired patients due to neurological diseases, such as stroke and amyotrophic lateral sclerosis [25,26]. Furthermore, we found that the performance of this acoustic-inspired decoder is driven by both cortical and subcortical signals.

## Methods

### Study participants

This study enrolled 4 participants—2 females and 2 males, aged 20, 25, 19, and 19 years for participants 1, 2, 3, and 4, respectively. They were undergoing epilepsy treatment at the Second Affiliated Hospital of Zhejiang University School of Medicine. As part of their treatment, they had sEEG electrodes surgically implanted (with the number of electrodes being 12, 8, 11, and 13 for 4 participants, respectively) to monitor epileptic seizures for 1 to 2 weeks. All participants were native Mandarin Chinese speakers, with the left hemisphere identified as the dominant hemisphere for language processing. Prior to data collection, all patients were informed of study procedure and signed informed consent form to participate. The study was approved by the Ethics Committee of the Second Affiliated Hospital of Zhejiang University School of Medicine.

### Task design

The design of this Mandarin Chinese BCI system is predicated on decoding the neural signals during the articulation process of Mandarin Chinese characters, ultimately converting them into Mandarin Chinese sentences. Hence, the tasks were specifically tailored for this purpose. We designed 2 distinct sets of tasks for training and evaluating the BCI system, respectively. The first set of tasks involves reading 407 unique Mandarin Chinese characters aloud. Although there are only 407 characters, they encompass all the phonetic syllables of Mandarin Chinese characters. The objective is to enable the system to decode virtually all Mandarin Chinese characters with as minimal training as possible, achieving a truly unrestricted Mandarin Chinese decoder. The second set of tasks, aimed at assessing the performance of the BCI system, employs Mandarin Chinese sentences of varying lengths as evaluating samples, to closely simulate the performance of this system in everyday life scenarios.

During the model training data collection phase (as depicted in Fig 1B, upper panel), participants were required to read 407 Mandarin Chinese characters corresponding to Mandarin Chinese syllables with tones, 3 to 5 times (3 to 5 trials per syllable × 407 syllables, depending on the monitoring duration for different participants). These 407 syllables encompass virtually all the pronunciations of Mandarin Chinese characters. Moreover, we designed the study to maintain as balanced a number of syllables as possible across the 4 tones, as shown in Fig. S1. To mimic natural speech patterns closely, each character was embedded within a carrier sentence by adding fixed characters before and after the target character. Thus, in every trial, participants read a complete sentence incorporating the designated syllable. For example, for the target syllable “ài”, the constructed sentence for reading was “
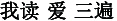
” ([ài], translating to “I read love three times”). All participants were pre-acquainted with the reading materials.

During the data collection process for evaluating the model (as illustrated in Fig 1B, lower panel), participants were required to read a total of 100 randomly selected common Mandarin Chinese sentences, with each word or sentence being read only once. These 100 samples were divided into 10 groups based on character length, ranging from 2 characters to more than 10 characters, with each group containing 10 different samples of the same character length. Participants were instructed to read aloud character by character at a normal pace, following prompts displayed on a monitor in front of them. Each character was allotted a reading time of 1.5 to 2 s, with a preparatory period of 0.3 to 0.5 s before reading each character. All evaluating data were collected within a single day.

### Data collection

#### Electrode anatomy localization and visualization

We utilized SPM12 for co-registering each participant’s preoperative T1 magnetic resonance imaging with their postoperative computed tomography (which includes electrode locations). Subsequently, we employed the FreeSurfer neuroimaging analysis software [27] to reconstruct the pial surface and determine the anatomical structure where each contact (channel) is located. The standard Montreal Neurological Institute template brain [28] was used for visualization in Fig. 1C.

#### Neural signal recoding and preprocessing

The electrode implantation plan, encompassing the location and number of implants, was devised solely for the treatment of epilepsy. Neural signals were recorded using a multi-channel electrophysiological recording device, specifically the Neurofax EEG-1200 produced by Nihon Kohden Corporation, Japan, and were recorded at a sampling rate of 2,000 Hz.

The neural signals collected were anti-aliased (low-pass filtered at 200 Hz) and notch filtered at 50, 100, 150, and 200 Hz to eliminate line noise, and channels exhibiting poor quality were excluded. Poor signals were defined as channels with a low signal-to-noise ratio or abnormal recordings identified during the inspection process. Signals are further down-sampled to 400 Hz. Neural signals were segmented according to task annotations, aligning them with the pronunciation process of individual characters.

To find speech-related channels, we compared the power spectral density (PSD; computed using Welch’s method) of neural signals from speech and silence parts within the same trial. For each channel (without channels located in white matter), a paired *t* test was conducted to compare the mean PSD values (4 to 150 Hz) of the speech condition (after acoustic onset) and silence condition (before acoustic onset). All resultant *P* values were corrected to control the false discovery rate (FDR) using the Benjamini–Hochberg procedure [29]. Finally, channels exhibiting a significant difference (*P* < 0.05) between speech and nonspeech conditions were selected. In addition, based on the reconstruction and localization results of all electrodes, certain channels located in the visual cortex (such as those situated in the occipital lobe) were also excluded. This exclusion was implemented to prevent visual-related signals from contributing to the decoding process. The locations of the channels ultimately selected for participation in the decoding process are displayed in Fig. 1C (63, 36, 30, and 66 selected channels for 4 participants, respectively), with the channels of the 4 participants represented in different colors.

#### Audio recording and preprocessing

Microphone recordings were obtained synchronously with the neural recordings using a directional microphone (manufactured by Audio-Technica) at 44.1 kHz. Given that the recording environment was situated within a hospital ward, temporary soundproof barriers were erected around the patient to ensure the quality of the recordings, aiming to isolate them from noise as much as possible (Fig. 1A). To prevent the recording of audio signals from contaminating the neural signals, we employed 2 entirely isolated sets of equipment to record these 2 types of data separately. Prior to utilizing these data, we calculated the correlation between audio signals and neural signals within the same time period (randomly selected 10-min intervals). The results confirmed that the recording of audio signals did not contaminate the neural signals (Fig. S2).

Initially, all audio recordings were manually audibly reviewed to identify and exclude samples with serious issues affecting recording quality, such as noticeable noise interference and character pronunciation errors. Correspondingly, the neural signals associated with these excluded audio samples were also removed. For training the syllable component prediction models, the start and end points of each character’s pronunciation are labeled. In addition, we also label the segmentation points of the initial and final sounds during the pronunciation process of each character to verify whether the temporal focus of syllable component prediction models aligns with the corresponding articulation process. In this study, we adopted a combination of automatic labeling using software and expert annotation. All audio materials were first automatically labeled using the PRAAT software [30], after which all labeling results were verified and finally confirmed by 3 phonetic experts.

### Analysis of audio signals

#### Acoustic feature extraction

In the design process of the 3 element prediction models, relevant acoustic features were utilized to enhance the prediction performance. Specifically, the initial prediction model employed acoustic–phonetic features, which are attributes inherent to the initials themselves, rather than being related to the recorded audio signals. Chinese is a tonal language, where speakers can modulate the pitch of their voice by adjusting the tension of their vocal cords to produce variations in pitch height and contour, thereby distinguishing different tones to convey various meanings. In this study, the pitch feature F0 was extracted from the audio signal and applied to the tone prediction model. The pronunciation characteristics of the final sounds can be assessed through 3 formants, with the first 2 formants being related to the position of the tongue, making them suitable for use in the final prediction model. Parselmouth [31], a Python implementation of PRAAT, was applied to obtain pitch and formant data [30].

#### Audio signal classification through support vector machine

In Fig. 3, various results of audio signal classification are presented, where panel (B) shows the outcomes of tone classification, panel (D) illustrates the classification of monophthongs, panel (E) displays the classification results for all vowels, and panel (G) reveals the classification outcomes for final clusters. All classifications were conducted using a one-against-all multi-class support vector machine (SVM) algorithm. The following decision function is employed for classifying $x_{\mathrm{new}}$:

fxnew=argmaxi∈1⋯M∑support vectorsytαtikxtxnew+bi (1)

where t represents the indexed sample and $i\in \left\{1,\cdots ,\;M\right\}$ denotes the total of M binary SVMs that need to be trained, involving the solution of M quadratic programming problems.

### Analysis of neural signals

#### Neural signal similarity analysis

**Fig. 2.**
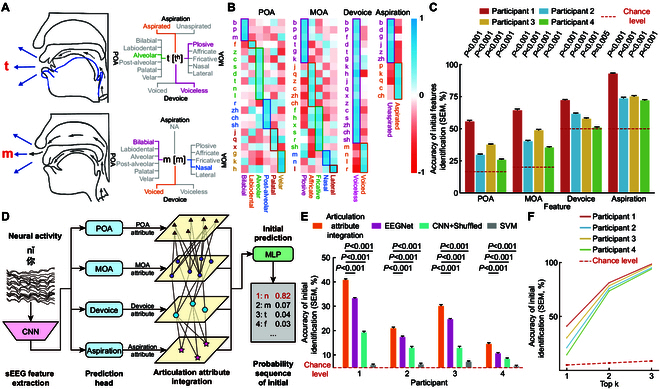
Design motivation and performance of initial prediction model. (A) Articulatory descriptions of the initials “t” and “m” characterize acoustic–phonetic features (on the left). Within the dynamic acoustics process, the gray section denotes the occlusive phase of acoustics, while the blue section indicates the release phase. The multiple acoustic–phonetic features are combined to describe these 2 phonemes (on the right). (B) The correlation between specific initials and the average of their corresponding category is visualized through a series of heat maps, where the color representing each initial is matched to the color of its category. (C) Prediction accuracy (mean ± SEM) of 4 initial features for 4 participants. (D) The initial prediction model utilizes a GNN to integrate various acoustic–phonetic features. This AAI approach effectively captures the complex interactions of voicing features to accurately map neural signals onto these feature spaces. (E) The prediction accuracy (mean ± SEM) of the initial model was measured for 4 participants and compared with scenarios where the acoustic–phonetic feature labels were shuffled (CNN + Shuffled), as well as with the EEGNet, SVM, and a baseline accuracy (Chance). (F) Probabilities (mean ± SEM) of the correct initial being included in the top 1, top 2, and top 3 probability sequence.

Fig. 2B displays the Pearson correlation between specific initials and the average of their corresponding category. We discuss 2 scenarios based on whether the initial is included in the corresponding category. In the first scenario, when the $i^{\mathrm{th}}$ initial does not belong to the $j^{\mathrm{th}}$ category of initials, we first calculate the average neural signal for all trials of the $i^{\mathrm{th}}$ initial and the average neural signal for all initials within the $j^{\mathrm{th}}$ category. The calculation of the correlation $R_{j}^{i}$ is as follows:

$$R_{j}^{i}=\text{corr}\left(\bar{\mu_{i}},\frac{\sum_{t=1}^{N_{j}}\bar{\mu_{t}}}{N_{j}}\right),i\notin {\text{Cluster}}_{j}$$ (2)

where corr represents the Pearson correlation between 2 sequences, and $N_{j}$ denotes the number of initials involved in the $j^{\mathrm{th}}$ category. In the second scenario, where the $i^{\mathrm{th}}$ initial belongs to the $j^{\mathrm{th}}$ category of initials, to exclude the positive bias introduced by self-correlation, we randomly divide the N trials of the $i^{\mathrm{th}}$ initial into 2 groups and calculate their average neural signals, $\bar{\mu }$ and $\tilde{\mu }$​, respectively. Then, we calculate the average of $\bar{\mu }$ for all initials within the $j^{\mathrm{th}}$ category. The calculation of the correlation $R_{j}^{i}$ is as follows:

$$R_{j}^{i}=\text{corr}\;\left(\tilde{\mu_{i}},\frac{\sum_{t=1}^{N_{j}}\bar{\mu_{t}}}{N_{j}}\right),i\in {\text{Cluster}}_{j}$$ (3)

#### Clustering of finals

We cluster similar finals together due to the difficulty of differentiation brought about by dynamic nature of compound final articulation. We consider a total of cluster number within the range of 10 to 15 to balance the granularity of classification and the classification accuracy. We employed the *k*-means clustering algorithm with silhouette coefficient to determine the optimal cluster number using the averaged formant features (F1, F2, and F3) across all audio samples of each final class as input, finding the optimal cluster number of 11. We then merge the finals within one cluster as one final cluster.

### Decoder performance evaluation

#### Character error rate

The character error rate (CER) is adopted as a metric to evaluate the performance of our proposed decoder, which is defined as follows:

$$CER_{i}=\frac{{\mathrm{EC}}_{i}}{N_{i}}$$ (4)

where $CER_{i}$ represents the character error rate for the $i^{\mathrm{th}}$ evaluating sample, ${\mathrm{EC}}_{i}$ denotes the number of characters in the $i^{\mathrm{th}}$ decoded result that do not match the ground truth, and $N_{i}$ indicates the total number of characters in the $i^{\mathrm{th}}$ evaluating sample. Naturally, the character-level accuracy (CLA) is defined as follows:

$${\mathrm{CLA}}_{i}=1-\frac{{\mathrm{EC}}_{i}}{N_{i}}$$ (5)

#### Syllable-level accuracy

During the evaluation of the performance of language model, syllable-level accuracy (SLA) is used to assess the model’s ability to convert syllables into Chinese characters. SLA is defined as follows:

$${\mathrm{SLA}}_{i}=\frac{{\mathrm{AS}}_{i}}{N_{i}}$$ (6)

where $SLA_{i}$ represents the syllable-level accuracy for the $i^{\mathrm{th}}$ evaluating sample, ${\mathrm{AS}}_{i}$ denotes the number of syllables in the $i^{\mathrm{th}}$ decoded result that match the ground truth, and $N_{i}$ indicates the total number of characters in the $i^{\mathrm{th}}$ evaluating sample.

#### Element-level accuracy

We adopt element-level accuracy (ELA) to evaluate the performance of the language model since it reflects the language model’s elements correction capability within a syllable. First, each Chinese character in the decoded word or sentence is converted into a syllable. Then, the initial, tone, and final components of each decoded syllable are extracted. We calculate ELA as follows:

$${\mathrm{ELA}}_{i}=\frac{1}{3}\cdot \frac{\sum_{j=1}^{n}\left(1\left(\text{initia}l_{j}\right)+1\left(\mathrm{ton}e_{j}\right)+1\left(\text{fina}l_{j}\right)\right)}{n}$$ (7)

where ${\mathrm{ELA}}_{i}$ represents the element-level accuracy for the $i^{\mathrm{th}}$ evaluating sample, nrepresents the total number of characters of the $i^{\mathrm{th}}$ evaluating sample, and $1\left(\cdot \right)\;$ is an indicator function with a value of 1 for a correct prediction of the corresponding syllable element and otherwise 0.

### Syllable element prediction model

#### Network architecture

We adopt a simple yet effective plain convolutional neural network (CNN) architecture as the backbone for syllable element feature extraction. The detailed architecture of the network is provided in Table S3. The subjects’ pre-processed sEEG of input channels C is first passed to a stem layer containing a one-dimensional (1D) convolution layer. The 1D convolution layer adopts a kernel size of K × 1, a stride of S, and an output channel number of 64. The numbers of the kernel size K and stride of S are adjusted for each subject. Each convolution block consists of a 1D convolution layer (followed by a batch normalization layer and a ReLU activation) and a max pooling layer. The number of the output channel of the first 2 blocks is set to 128, while the number of the last 2 blocks is set to 256. Then, we adopt a global average pooling layer and a fully connected layer to map the output of the last block to fit the prediction targets of different syllable elements.

#### Initial prediction model

For the initial prediction task, we follow the same experimental setup described in the “Experimental setup” section and report the mean and standard deviation of the top 1 accuracy for both initial prediction and each acoustic–phonetic feature. Let $z\in {\mathbb{R}}^{c_{\bar{f}}\times T}$ be the feature extracted by the CNN backbone, where $c_{\bar{f}}$ denotes the channel dimension and T denotes the temporal dimension of the feature, respectively. On top of the extracted feature z, we first utilize a 2-layer multilayer perceptron (MLP) prediction head with a hidden dimension of 256 to map the flattened z to a neural representation $\tilde{z}\in {\mathbb{R}}^{c_{f}}$, where $c_{f}$ denotes the hidden dimension of the mapped neural representation $\tilde{z}$.

Inspired by the articulation of each Mandarin initial consonant, we propose the articulation attribute integration (AAI) module to better capture the acoustic–phonetic features during the articulation process. The acoustic–phonetic features include the place of articulation (POA), manner of articulation (MOA), aspiration, and devoice. These acoustic–phonetic features can be considered as 4 attributes associated with each initial consonant. We first decompose the initial prediction problem into 4 attribute categorization subproblems. In particular, we utilize 4 learnable prototypes—$W_{\mathrm{poa}}\in {\mathbb{R}}^{c_{f}\times d_{\mathrm{poa}}}$, $W_{\mathrm{moa}}\in {\mathbb{R}}^{c_{f}\times d_{\mathrm{moa}}}$, $W_{\mathrm{asp}}\in {\mathbb{R}}^{c_{f}\times d_{\mathrm{asp}}}$, and $W_{\mathrm{dev}}\in {\mathbb{R}}^{c_{f}\times d_{\mathrm{dev}}}$—to map the neural representation $\tilde{z}$ to representations in the acoustic–phonetic attribute spaces. Here, $d_{\mathrm{poa}}$, $d_{\mathrm{moa}}$, $d_{\mathrm{asp}}$, and $d_{\mathrm{dev}}$ represent the cardinality of the POA, MOA, devoice, and aspiration attributes, respectively. We concatenate the prototypes and perform attribute aggregation by multiplying with an adjacency matrix $\tilde{𝔸}$:

$$\begin{array}{c} \begin{array}{c} \bar{𝕎}=\text{CONCAT}\left(W_{\mathrm{poa}},W_{\mathrm{moa}},W_{\mathrm{asp}},W_{\mathrm{dev}}\right)\\ \tilde{𝕎}=\text{ReLU}\left(\bar{𝕎}\cdot \tilde{𝔸}\right) \end{array} \end{array}$$ (8)

where CONCAT denotes the concatenation operation and $\bar{𝕎}\in {\mathbb{R}}^{c_{f}\times d}$, where $d=d_{\mathrm{poa}}+d_{\mathrm{moa}}+d_{\mathrm{asp}}+d_{\mathrm{dev}}$. The adjacency matrix $\tilde{𝔸}$ is defined by adding self-loop as $\tilde{𝔸}=𝔸+𝕀$, where $𝕀\in {\mathbb{R}}^{d\times d}$ is the identity matrix. ${𝔸}_{i,j}=1$ if the attribute i and attribute j coexist in the onset process of any initial consonant and ${𝔸}_{i,j}=0$ otherwise. Notice that 𝔸 is only initialized by the previous condition and updated during training. The aggregated prototypes $\tilde{𝕎}$ are then multiplied with acoustic–phonetic representation $\tilde{z}$ to predict the 4 attribute labels. A 2-layer MLP prediction head with a hidden dimension of 256 is utilized to predict the initial label. The overall loss function is defined as follows:

$$L=\alpha \cdot L_{\text{attr}}+L_{\text{init}}$$ (9)

where $L_{\text{attr}}$ and $L_{\text{init}}$ stand for categorical cross-entropy loss for attribute classification and initial classification, respectively. α is a scaler to balance the 2 losses.

#### Tone and final cluster prediction model

In the field of speech processing, the key factor in tone perception relies on pitch-related features where the fundamental frequency (F0) plays a crucial role. Similarly, vowel formants, particularly the first 3 formants (F1, F2, and F3), are essential acoustic parameters that represent the resonant frequencies of the vocal tract. These formants are directly influenced by articulatory features, with tongue position playing a central role [32]. F1 reflects tongue height, where a higher tongue position results in a lower F1, while a lower tongue position leads to a higher F1. F2 corresponds to tongue frontness or backness; a fronted tongue increases F2, whereas a retracted tongue decreases it. F3 is influenced by both tongue tip configuration and lip rounding, with a lowered tongue tip or rounded lips generally reducing F3. The similarity of formant features among different finals serves as a valuable indicator for measuring the articulation process, which we aim to decode using sEEG. Besides directly predicting the tone and final using the features extracted from CNN, we introduce a neural-audio regularization (NAR) module, which enhances the CNN’s ability to model the similarity of the articulation process by using formant features as guidance. Let Z be the extracted feature from a training batch of sEEG data, and Fdenotes the corresponding formant features derived from the associated audio data. For tone prediction, we specifically utilize the F0 feature as the formant representation, whereas for final prediction, we incorporate the F1, F2, and F3 features. Subsequently, we calculate the pairwise neural Euclidean distance $D_{i,j}^{n}$ and the formant Euclidean distance $D_{i,j}^{f}$ based on the extracted neural feature Z and formant feature F, respectively. To measure similarity, we employ the Student’s *t* distribution, defined as follows:

$$S_{i,j}=\frac{\Gamma \cdot \left(\frac{\nu +1}{2}\right)}{\sqrt{\nu \pi }\cdot \Gamma \cdot \left(\frac{\nu }{2}\right)}\cdot {\left(1+\frac{D_{i,j}^{2}}{\nu }\right)}^{-\frac{\nu +1}{2}}$$ (10)

where Γ and ν refer to the gamma function and the degree of freedom, respectively. D denotes the Euclidean distance. The objective function to train NAR is defined to be the logistic loss between $S_{i,j}^{n}$ and $S_{i,j}^{f}$ derived from $D_{i,j}^{n}$ and $D_{i,j}^{f}$ , respectively

$$L_{\mathrm{NAR}}=-{𝔼}_{i,j}\cdot \left[S_{i,j}^{a}\cdot \log\frac{S_{i,j}^{a}}{S_{i,j}^{n}}+\left(1-S_{i,j}^{a}\right)\cdot \log\frac{\left(1-S_{i,j}^{a}\right)}{\left(1-S_{i,j}^{n}\right)}\right]$$ (11)

The overall loss function is defined to be a weighted sum of $L_{\mathrm{NAR}}$ and the corresponding categorical cross-entropy loss for either tone or final prediction.

#### Experimental setup

The experiments were conducted using PyTorch [33] on NVIDIA V100 GPUs. For investigating syllable element-level prediction, the data from 3 trials were combined and divided into 80% for training and 20% for evaluating, using 20 distinct random seeds. Additionally, to demonstrate the robustness of the proposed approach, we provide evaluation metrics based on a 5-fold cross-validation protocol in Fig. S3. This setup facilitated the calculation of mean and variance for performance metrics. We carry out experiments on all 4 subjects with the same experimental setup. For each subject, 20% of the training data from the first repetition were allocated for tuning size K and stride of S. The specific choices of kernel size and stride for each subject are detailed in Table S4. The models were trained using the AdamW optimizer, with a base learning rate of lr=0.0003. Momentum parameters are set to $\beta_{1}=0.9$, $\beta_{2}=0.009$, and a cosine decay approach was adopted for the learning rate schedule. To mitigate overfitting, a dropout layer with a rate of 0.3 is applied after the max pooling layer in each convolutional block. The training was conducted over 80 epochs, incorporating an early stopping criterion based on a 5-epoch non-improvement threshold on the validation set. The complete list of parameters used for model training is available in Table S5. For sentence-level performance assessment, syllable element prediction models were trained on the entire dataset for each subject over a fixed epoch number of 50.

#### Element prediction with baseline predictors

In this study, we employed SVM classifiers and the EEGNet [34] as baseline performance for syllable element prediction. To ensure consistency across comparisons, we followed the same data split strategy and evaluation metrics described in the “Experimental setup” section. For training the SVM classifiers, we utilized a radial basis function (RBF) kernel. The regularization parameter and kernel parameter gamma were optimized through a grid search using 5-fold cross-validation on the training set for each random seed. Additionally, we adopted the one-versus-the-rest (OvR) strategy for multiclass classification to handle multiple syllable element categories effectively. For training EEGNet, we adhered strictly to the original network architecture described in its reference paper. Specifically, the number of temporal filters and spatial filters was set to 4 and 2, respectively.

### Language modeling

**Fig. 3.**
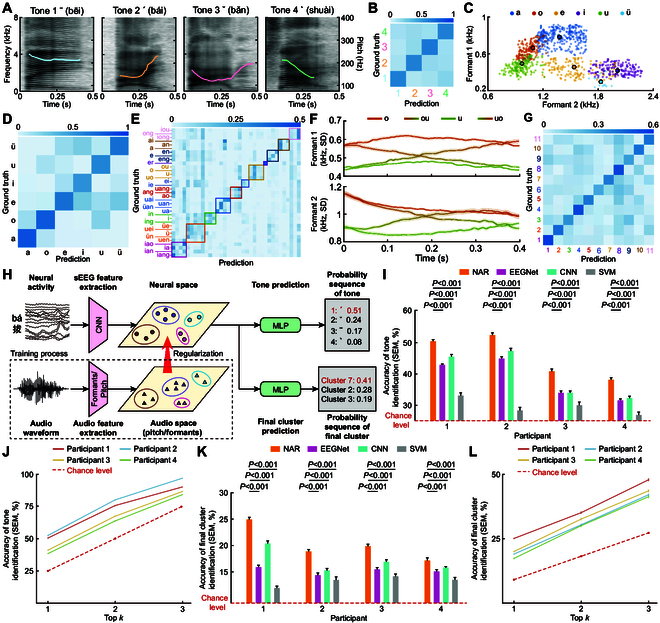
Design and performance of tone and final cluster prediction models. (A) Examples of the display of Mel-frequency spectrograms and pitch curves for the 4 tones, with data sourced from participant 1. (B) Based on pitch feature extracted from audio signal, an SVM model was used to classify the 4 tones (participant 1). (C) Visualization results of the formant features for 6 single finals. Since the formants are relatively stable during the articulation of single finals, the average value of the entire process is used to represent the corresponding formant feature. Dots of the same color represent different samples of the same single final, with the larger dot in the corresponding color representing the average of all the dots. (D) Based on formant features extracted from audio data, an SVM model was used to classify the 6 single finals (participant 1). (E) Based on formant features extracted from audio data, an SVM model was used to classify the 35 finals (participant 1). Finals of the same color were clustered into the same category using the *k*-means clustering method. (F) The dynamic changes of formant 1 and formant 2 during the articulation process of 4 phonetically similar finals (“o”, “u”, “ou”, and “uo”) are depicted (mean ± SEM). (G) Based on formant features extracted from audio data, an SVM model was used to classify the 11 final clusters (participant 1). (H) Model design for tone and final cluster prediction. This approach aligns the distribution of sEEG data in the neural space with the audio data distribution, especially in terms of pitch and formant features, to improve the extraction of neural features for tone and final cluster categorization. Particularly, we employed a NAR term, based on similarity measurements, alongside the categorical cross-entropy loss during the training of the model. (I) The prediction accuracy (mean ± SEM) for tone was measured across 4 participants and compared to scenarios without neural-audio regularization (CNN), as well as with EEGNet, SVM, and a baseline accuracy (Chance). (J) Probabilities (means ± SEM) of the correct tone being included in the top 1, top 2, and top 3 prediction results for each of the 4 participants. (K) The prediction accuracy (mean ± SEM) for final cluster was measured across 4 participants and compared to scenarios without neural-audio regularization (CNN), as well as with EEGNet, SVM, and a baseline accuracy (Chance). (L) Probabilities (means ± SEM) of the correct final cluster being included in the top 1, top 2, and top 3 prediction results.

Given a sequence of syllable element predictions, denoted as $\varepsilon ={\left\{\left(i_{i}^{k},\;t_{i}^{k},\;f_{i}^{k}\right)\right\}}_{i=1}^{N}$, a language model is designed to output the most probable sentence, where *N* represents the total number of characters in the sentence. The elements $i_{i}^{k},t_{i}^{k},f_{i}^{k}$ represent the top *k* predictions for the initial, tone, and final elements of the $i^{\mathrm{th}}$ character, respectively. The model’s top 3 accuracy for tone and initial predictions, as shown in Fig. 3J and Fig. 2F, respectively, reveals that the 3 most probable predictions often correctly identify these elements. Our approach involves constructing a 5-gram language model that evaluates the probability of initial and tone sequences, denoted as ${\left\{\left(i_{i}^{3},\;t_{i}^{3}\right)\right\}}_{i=1}^{N}$, using the top 3 predictions for each. The training of this 5-gram model utilized CLUECorpus2020 [35], a comprehensive Mandarin Chinese corpus containing 35 billion Chinese characters retrieved from Common Crawl. CLUECorpus2020 contains a wide range of topics, including news, entertainment, sports, health, international affairs, movies, celebrities, and more. To develop our 5-gram model, we randomly selected 20% of this training set and implemented the model using KenLM [36]. Instead of employing a character-based vocabulary, we have constructed a specialized vocabulary comprising pairs of initials and tones. This approach results in a vocabulary of 84 distinct elements, derived from 21 initials combined with 4 different tones. We then utilize the Google Pinyin application programming interface to convert the initials from the top 50 probability sequences into Mandarin characters, forming sentences. For each initial sequence, we extract 2 sentence suggestions, thereby cumulatively creating a total of 100 sentences.

The majority of current Pinyin input methods do not rectify incorrect or illogical initial sequences. Instead, they endeavor to construct a sentence from the input, often resulting in combinations of characters that resemble sentences but lack coherent, natural language meaning. Consequently, it is advantageous to utilize large language models (LLMs) to determine the most probable sentence out of 100 potential options generated, harnessing the LLM’s capacity to discern coherent and meaningful language from a multitude of sentence possibilities. In our approach, we utilize the Chinese-LLaMA-7B model [37] to evaluate the likelihood of different sentence options. Perplexity PPL of each sentence candidate is calculated using the following equation:

$$\mathrm{PPL}\left(\cdot \right)=e^{-\sum_{i=1}^{N}\log P\left({\mathrm{ch}}_{i}|{\mathrm{ch}}_{1},\;{\mathrm{ch}}_{2},\cdots ,\;{\mathrm{ch}}_{i-1}\right)}$$ (12)

where $P\left({\mathrm{ch}}_{i}|{\mathrm{ch}}_{1},{\mathrm{ch}}_{2},\cdots ,{\mathrm{ch}}_{i-1}\right)$ represents the probability assigned by the LLM to the character ${\mathrm{ch}}_{i}$ based on the sequence of preceding characters. We then select the sentence with the lowest perplexity as the final output. This method ensures that the output is a grammatically correct sequence of characters and a coherent and contextually appropriate sentence.

### Contribution of different anatomical brain regions

We analyze the contribution of brain regions from 2 distinct perspectives. The first perspective utilizes sEEG signals from single functional brain regions as system inputs for Mandarin Chinese decoding, where the decoding performances of these regions serve as the metric for assessing their contribution level. The second perspective involves using all channels as model inputs, where we determine the contribution of brain regions based on the significance level of different channel signals in the decoding decision channel saliency calculated by class activation maps [38].

#### The decoding accuracy of different brain regions

We train syllable element prediction models utilizing sEEG signals of channels that cover the specific brain regions that we aim to analyze and report the prediction performance. We follow the same experimental setup as described in syllable element prediction model and evaluation part. This method allowed for a focused investigation of each region’s role in the decoding process from the perspective of the prediction performance.

#### The contributions of electrodes located in different brain regions to decoding

Channel saliency score is calculated by class activation maps [38]. Given an sEEG signal $x_{i,j}\in {\mathbb{R}}^{T\times C}$, where T is the length of the signal and C is the total number of channels, we calculate a channel saliency score ζ:

$$\zeta \left(c\right)=\frac{1}{T}\cdot \sum_{i=1}^{T}\text{ReLU}\left(\frac{\partial_{y}}{\partial_{x_{i,c}}}\right)$$ (13)

where y is the one-hot logit vector corresponding to the true label of the underlying prediction task, and ReLU is the activation function. As illustrated in Eq. (6), the partial derivative is calculated with respect to the input sEEG signal and averaged over the time dimension. This measure indicates how changes in the information content of channel c influence the accurate prediction of the true label. We then normalize the saliency score $\zeta \left(c\right)$ for each channel c to be between zero and one. The second measurement provided a comprehensive understanding of how the change of information from specific brain regions affects the Mandarin Chinese decoding process, highlighting the contribution level of each channel when using all-channel sEEG for language decoding.

#### Model temporal saliency of syllable prediction models

Similar to the calculation of channel saliency score in Eq. (12), we calculate the temporal saliency score $\eta \left(t\right)$:

$$\eta \left(t\right)=\frac{1}{C}\cdot \sum_{i=1}^{C}\text{ReLU}\left(\frac{\partial_{y}}{\partial_{x_{t,i}}}\right)$$ (14)

where $x_{t,i}$ refers to sEEG signal of channel i at time stamp t. Different from Eq. (13), the partial derivative is averaged over the channel dimension.

## Results

### Overview of decoder design

A 3-phase (channel selection, syllable prediction, and language modeling) decoder was proposed for decoding the neural signal to the complete sentences (Fig. 1D). For each participant, we used signal from the selected channels associated with speech and excluded those located in visual cortex and white matter (Fig. 1C, details of channel selection in Methods). During the syllable prediction phase, 3 CNNs were trained to extract features representing the initials, tones, and finals from the preprocessed sEEG signal segments of the syllables being spoken. For each syllable, we aligned the tone and final neural features with acoustic features extracted from the corresponding audio signal using NAR. Initial features were mapped into articulatory features, with their relationships further refined by a graph neural network (GNN). In the evaluating process of decoding complete sentences, we used the above 3 trained models to predict initial, tone, and final syllable by syllable, and finally formed a probability sequence. In the third phase, a language model was established to output the most probable sentence based on the probability sequence of predicted elements of all syllables. The language model was trained on encyclopedia-style question–answer dialogues retrieved from Common Crawl. The probability sequence of top 3 initials, top 3 tones, and the leading final cluster (one final cluster contains several finals with similar acoustic characteristics) forms the output of the 3 syllable element prediction models. Furthermore, the language model is capable of translating syllable sequences into Mandarin sentences, correcting errors in syllable element predictions, and enhancing the overall performance of sentence outputs.

### Performance of the brain-to-sentence decoder

To fully illustrate the decoding capability of this system in the natural Mandarin environment, we randomly selected sentences for daily use ranging in length from 2 characters to more than 10 characters, taking 10 of each length for a total of 100 test samples. First, we analyzed the distribution of the average character error rate for each sentence (Fig. 1E). Table S1 presents examples of decoded sentences across various character error rate levels. For participant 1, the median character accuracy is 71.00%, with 30.00% of the decoded sentences being completely accurate (Fig. 1F). The decoded sentences exhibit a low error rate and high intelligibility, making them both meaningful and viable for practical applications.

We calculated the character-level decoding accuracy and found that the average accuracies for the 4 participants were 66.62%, 51.37%, 46.34%, and 47.56%, respectively (Fig. 1G and Fig. S4). Notably, sentences of moderate length (about 4 to 8 characters) yielded higher accuracies. Conversely, the accuracy for decoding shorter phrases of 2 to 3 characters was lower, primarily due to the lack of sufficient linguistic context. This limitation hindered the model’s ability to distinguish between phrases with identical pronunciations but different meanings, such as “
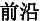
” and “
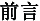
”, with the same syllable (qián yán) and different meanings (frontier and preface, respectively). We verify that the generalization performance of the model is strong, and it can solve the parts not covered by the training data in tens of thousands of Mandarin Chinese characters (Fig. 1H), which also highlights the effectiveness of our acoustic inspired decoding strategy.

### Syllable prediction of initial

There are a total of 21 distinct initials in the Chinese character syllables (Table S2). The articulation of initials is produced by overcoming the obstruction of airflow as it passes through the oral or nasal cavity. Four features—POA (including bilabial, labiodental, alveolar, retroflex, alveolo-palatal, and velar), MOA (including plosive, affricate, fricative, nasal, and lateral), devoice (including voiced and voiceless), and aspiration (including unaspirated and aspirated)—uniquely describe the articulation process of each Mandarin initial (Table S2). Fig. 2A shows the articulation process and corresponding articulation features of 2 initials, which is “t” ([t^h^]) and “m” ([m]).

To assess the similarity in neural activity during the articulation of initials sharing common features, we analyzed the correlation between specific initials and the average of their corresponding category (Fig. 2B). Our findings reveal a positive correlation for POA (*r* = 0.52), MOA (*r* = 0.54), devoicing (*r* = 0.49), and aspiration (*r* = 0.59). This correlation is visually represented by 4 diagonal lines across the heatmap for each feature. Moreover, the correlation is significantly higher than that with irrelevant articulation features (*P* < 0.001 for POA, MOA, devoice, and aspiration), indicating that the sEEG signals capture distinct information related to categorical articulation features. To further explore this, we developed classification models to decode these 4 articulatory features from the sEEG signals of each initial’s trial (Fig. 2C). The classifiers demonstrated that the sEEG signals contain important information about these features, with decoding accuracies substantially surpassing the chance level for all participants.

Building upon these insights, we design the initial prediction model by identifying the acoustic–phonetic features (Fig. 2D, details in Methods). We first map the neural signals to representations in the acoustic–phonetic feature spaces of POA, MOA, aspiration, and devoice. Given that the voicing of initials results from the interactions among diverse voicing features, we address this inherent relationship with a GNN as an AAI. As a result, the accuracy rates from the model are significantly above the chance level for the 4 participants (*P* < 0.001, Fig. 2E), achieving rates of 41.07%, 21.10%, 30.32%, and 14.65%, respectively, where the chance level is 5%. However, the sole prediction result from the model is evidently insufficient in accuracy for input into the language model for sentence transformation. Therefore, we calculated the accuracy considering multiple candidate outputs from the model. Notably, there was a significant increase in prediction accuracy as the number of candidate results grew (*P* < 0.001). In the top 3 most probable predictions, the probability of containing the correct initial for the 4 participants was 99.24%, 97.12%, 98.39%, and 95.03%, respectively (Fig. 2F). In summary, the initial prediction model can identify accurate initial results in most trials based on sEEG signals from selected channels, and its near-perfect top 3 accuracy narrows the possible initial candidates from 21 down to 3. This reduces the computational burden on the third phase of the decoder and enhances the efficiency of the selection process of language model.

### Syllable prediction of tone

Tone, a crucial aspect of Mandarin syllables, differentiates both lexical and grammatical meanings through variations in pitch carried by the finals [13,39]. We visualized the pitch changes over time for the 4 Mandarin tones (Fig. 3A), highlighting their distinct patterns and stability across trials of the same tone. To distinguish these tones quantitatively based on pitch variations, we employed an SVM classifier trained on pitch data from all trials, regardless of the rhymes on which the tones were borne. The 4 different tones can be distinguished from the perspective of pitch variations, and the values on the diagonal are significantly higher than the off-diagonal values in Fig. 3B (*P* = 0.017). The final classification accuracy was 57.51%, significantly above the chance level (*P* < 0.001). Consequently, we incorporated pitch into the training process of the tone prediction model (Fig. 3H, details in Methods). Aligning the distribution of sEEG data in the neural space with the distribution of audio data, particularly concerning pitch features, enhances the extraction of neural features for tone categorization. We incorporate a NAR term based on similarity measurement in addition to the categorical cross-entropy loss during model training. Audio signals will solely be utilized in the decoder training process and not during evaluating.

The incorporation of NAR significantly enhanced the performance of neural decoding models, yielding prediction accuracies of 50.60%, 52.75%, 41.19%, and 38.48% for the 4 participants, respectively, all markedly above the chance level (*P* < 0.001 for all, as shown in Fig. 3I). These accuracies also surpassed those of models trained without NAR, highlighting NAR’s importance. Given that a single prediction may not capture all correct tones, we adopted a strategy of selecting the top 3 most probable tones for each prediction. This adjustment significantly improved the accuracy of the final sentence predictions, with the correct tones being included in the top 3 predictions with probabilities of 90.77%, 97.66%, 87.21%, and 84.76% for the participants, respectively (Fig. 3J). Importantly, this probabilistic information about the tones is also conveyed to the language model, providing it with richer reference data for more accurate sentence generation.

### Syllable prediction of final

Mandarin syllables comprise 6 simple (a, o, e, i, u, and ü) and 29 compound finals (iou, ong, iong, ai, an, en, eng, er, ou, uo, ei, ie, uang, ang, ao, uai, uan, üan, ua, in, ing, üe, uei, ün, uen, ia, iao, ian, and iang), whose articulation involves vocal cord vibration and nuanced tongue positioning. The frequency spectrum’s formant features, F1 and F2, correspond to the tongue’s vertical and anteroposterior positions, respectively. We visualized the formant features F1 and F2 for all trials of 6 simple finals, revealing a clear clustering effect (Fig. 3C). An SVM classification model trained on F1 and F2 successfully differentiated the audio of the 6 simple finals, achieving a classification accuracy of 66.35%, significantly surpassing the chance level (*P* < 0.001; Fig. 3D).

However, once the compound finals were incorporated, the classification results deteriorated (15.46%, compared to the chance level, *P* = 0.14; Fig. 3E). This decline in performance can be attributed to the dynamic nature of compound final articulation, which involves transitioning from one articulatory shape to another. For instance, we visualized the changes in F1 and F2 during the articulation of the simple finals “o” and “u” alongside the compound finals “ou” and “uo” (Fig. 3F). The compound final “ou” is articulated by transitioning from the sound of “o” to “u”, while “uo” undergoes the opposite transition, making their articulatory configurations exceedingly similar and hence challenging to distinguish. Therefore, based on the resonance peaks of the finals in the audio, we employed the *k*-means algorithm to cluster finals with similar articulatory configurations, ultimately forming 11 distinct final clusters (Fig. 3E). The SVM classification models were used to identify the final clusters, achieving an accuracy of 40.86%, which is significantly higher than the chance level (*P* = 0.013, Fig. 3G).

Extracting the formant features from audio has proven beneficial for classifying final clusters. Therefore, we also employed the model used to predict tones with NAR to predict the final clusters based on sEEG signals (Fig. 3H, details in Methods). Despite the finals’ complexity, the model demonstrated accuracies of 25.10%, 19.02%, 20.04%, and 17.33% across participants, all statistically significant against the chance level. As the number of candidate clusters increased, the probability of including the correct final also rose (Fig. 3L). The inclusion of multiple finals within a single predicted cluster aimed to reduce the selection burden in the decoder’s third phase, acknowledging that the precision of initials and tones predominantly conveys the necessary information for accurate sentence prediction in Mandarin, thus mitigating the impact of less accurate final predictions.

### Language modeling

In this study, the language model is tasked with selecting the appropriate components to form syllables from the probability sequences of the 3 syllable elements (initials, tones, and finals). Subsequently, it was required to identify the most appropriate Mandarin Chinese characters matching each syllable’s pronunciation, thereby constructing sentences that are both semantically and grammatically coherent.

**Fig. 4.**
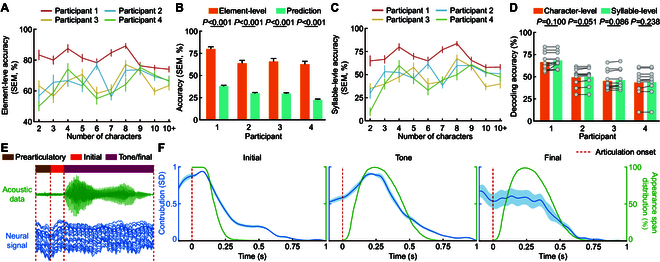
The analysis of language model and 3 syllable element prediction models. (A) Element-level accuracy (mean ± SEM) of sentences composed of different lengths for 4 participants. (B) Comparison between element-level accuracy (mean ± SEM) and the accuracy (mean ± SEM) of 3 element prediction models decoding along (Prediction). (C) Syllable-level accuracy (mean ± SEM) of sentences composed of different lengths for 4 participants. (D) Comparison between character-level accuracy (mean ± SEM) and syllable-level accuracy (mean ± SEM). The gray dots represent the accuracies of sentences in different lengths (ranging from 2 to 10+ characters, with a total of 10 different conditions), where the significance comparison is conducted using paired *t* tests. (E) Acoustic data and neural signal of articulation process of fěn. The articulation process is divided into 3 periods by phonetic experts. The first is the pre-acoustic period (100 ms). The second is the initial articulation period, which is generally shorter in length. The last is the articulation process of tones and finals. Because the tones are carried on the finals, the pronunciation process overlaps. According to the theory of phonetics, the articulation process of initials and finals overlaps. This study divides them according to the average position in the overlapping part. (F) Temporal saliency on neural signals of 3 syllable element prediction models used to identify the initial, tone, and final clusters, respectively, over the entire duration of a syllable (blue line, mean ± SD). The green line indicates the appearance span distribution with respect to time for the initial, tone, and final of all 407 syllables, with the start and end points marked by experienced phoneticians.

To enhance the accuracy of this process, we determined that it was necessary to input the top 3 initials, top 3 tones, and the first final cluster as element possibility sequences into the language model in the decoder. We calculated the accuracy of the syllable elements converted from the final output sentences (element-level accuracy), where each element is selected by the language model from the probability sequences. The element-level accuracy for the 4 participants (Fig. 4A) are 80.98%, 66.82%, 64.98%, and 63.80%, respectively, which are significantly higher than the top 1 accuracy of the 3 element prediction models (Fig. 4B). From the aforementioned results, it is evident that the language model plays a vital role in selecting the correct elements and correcting erroneous ones, thereby mitigating the accuracy demands placed on the brain signal decoding component.

Furthermore, to evaluate the language model’s proficiency in transforming syllable sequences into accurate character sequences through semantic integration, we computed the syllable-level accuracy and compared it with the character-level accuracy. The syllable-level accuracies for the 4 participants were 68.93%, 50.52%, 46.68%, and 44.50%, respectively (Fig. 4C), which closely aligned with the character-level accuracies (Fig. 4D). This convergence indicates that the language model adeptly translated nearly all correct syllables into their accurate character counterparts. The few observed instances of homophone conversion errors, such as “
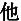
” (tā, [t^h^a], he) and “
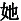
” (tā, [t^h^a], she), did not detract from the overall comprehension of the sentences for native Mandarin speakers. The ability of the language model to convert the pronunciation of characters and integrate context plays a decisive role in accurately translating syllable sequences, enabling it to select the correct characters from a vast array of homophones.

### Model attention across time

Acknowledging the challenges posed by the brief and often ambiguous transitions between the initial and final parts of syllables (Fig. 4E), our study approaches syllable decoding by treating the neural signals of the entire articulation process, including a 100-ms pre-acoustic period, as an integrated unit. This method allows us to preserve crucial information that might otherwise be lost during segmentation. We investigate if the temporal focus on the brain signals of the syllable component (initial, tone, and final) decoding models aligns with the vocalization processes of these components as reflected in the corresponding speech signals by calculating the temporal saliency of the syllable element prediction models.

Our analysis, illustrated in Fig. 4F (first panel for initial and mid panel for tone), shows a strong correlation between the temporal saliency on brain signals and appearance span distribution with respect to time of these syllable components in terms of audio data. This suggests that our models can effectively identify and capture syllable element relevant brain signal segments throughout the articulation process for accurate prediction. Compared to the initial and tone prediction models with prominent temporal focus regions, we observed a more uniform distribution in the saliency distribution for the final prediction model (Fig. 4F, last panel). This indicates that the final prediction model struggles to pinpoint final-related brain signal segments, which, in turn, might explain the model’s comparatively lower performance in predicting finals accurately.

### Contributions of different brain regions

**Fig. 5.**
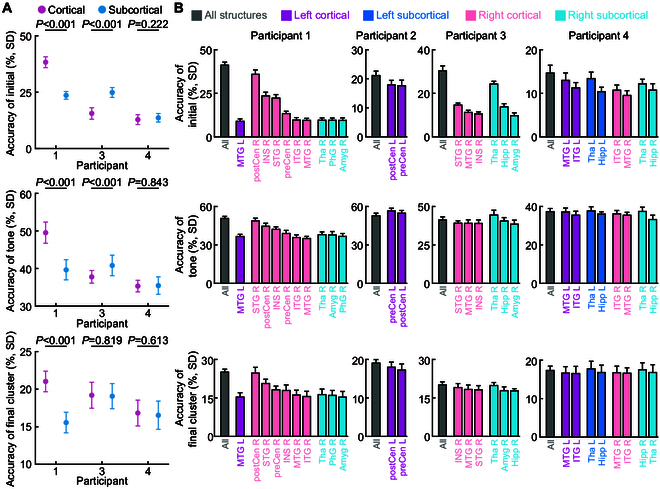
The decoding accuracy and contributions of each anatomical area. (A) Prediction accuracy of initial, tone, and final cluster using neural signals from cortical and subcortical brain structures. Accuracy data are means ± SD, and the results were presented for only 3 participants (1, 3, and 4), as no chosen electrodes of participant 2 were in the subcortical brain structures. (B) Prediction accuracy (means ± SD) of initial, tone, and final cluster using neural signals from different anatomical areas in cortical and subcortical structures of right and left hemispheres of the brain. The regions of the brain where the electrodes were located were different for 4 participants. Only the results of some important language-related anatomical area are selected to show. STG, superior temporal gyrus; MTG, middle temporal gyrus; ITG, inferior temporal gyrus; postCen, postcentral gyrus; preCen, precentral gyrus; INS, insula; Tha, thalamus; Hipp, hippocampus; Amyg, amygdala; PhG, parahippocampal gyrus.

In this study, we attempted to independently utilize signals from cortical and subcortical brain regions to predict 3 syllable elements. Comparative results at the element level were consistent, yet notable variances at the individual participant level emerged (Fig. 5A), indicating that the decoding efficacy is highly dependent on the specific brain regions engaged and the electrode placement. Participant 1 showed enhanced decoding performance with cortical signals, whereas participant 3 demonstrated better outcomes with subcortical signals. The results for participant 4 indicated no significant difference between the decoding effectiveness of the signals from these 2 regions. Overall, this suggests that while cortical regions are influential in speech decoding, the contribution from subcortical regions is also meaningful and should not be overlooked.

To further visualize speech-related information encoded in neural signals from different brain regions, we used signals from the electrodes located in the same regions as input to separately predict the initial, tone, and final cluster. Fig. 5B shows the prediction accuracy of 3 syllable elements from neural signals in different brain regions for 4 participants. Due to the variation in the brain regions and the number of electrodes implanted in different participants, cross-participant comparisons are not feasible. Results from patient 1 indicate that within the cortical areas, the superior temporal gyrus (STG), insula (INS), and the ventral sensorimotor cortex (composed of the postcentral gyrus and precentral gyrus, abbreviated as vSMC) exhibit higher decoding accuracies. This observation aligns with traditional theories of brain region function in speech production [18,39] and speech perception [19,20,40–42]. Within the subcortical areas, we observed that the thalamus exhibited significantly higher accuracy in certain prediction tasks (such as the initial prediction for participant 3) compared to other brain regions. Previous research has reported the involvement of the thalamus in human language processing [43,44]. Our findings further reveal that the thalamus is also engaged in human speech processing.

### Channel contribution

In the first phase of the decoder, the signals from all regions related to speech are utilized for language decoding. To determine their contribution in the trained model, we compute a saliency score for each channel as indication of contribution for syllable components prediction (Fig. S5). Subsequently, we also perform a correlation analysis with the decoding accuracy obtained using channels from individual brain regions as input data. The correlation between the prediction accuracy for 3 syllable elements and their contribution in 3 prediction models were found to be significant across all 4 patients (Fig. S6).

## Discussion

We tackled the challenges posed by the complexity of logosyllabic languages in speech BCI by taking the speech of individual character syllables as the decoding unit, achieving the offline transformation of speech-related brain signals into sentences for Mandarin Chinese with a promising performance. We show that a training dataset that includes all 407 valid syllables in Mandarin suffices for decoding the full Chinese character set when combined with a suitable language model. Additionally, we used monosyllable with tone as the modeling unit because previous unit selection study has proved that the use of syllable with tone effectively increases the speech recognition accuracy of Mandarin Chinese compared to other units such as characters or phonemes [45]. Since around 60% to 70% of the world’s languages are tonal [46], which is not reflected in the mainstream research on speech BCIs, our study of Mandarin Chinese contributed fundamental insights to the broader landscape of tonal language interface design and brain–computer system development.

Recently, numerous neural language decoding systems have incorporated language models as a component of their architecture [7,9]. In this study, we have developed a language model suitable for Mandarin Chinese speech BCI systems by integrating Pinyin [47] input, a widely adopted phonetic representation system based on the Latin alphabet with an *N*-gram model [48]. Pinyin syllable has been developed to facilitate literacy and serve as a bridge between Mandarin pronunciation and characters [49]. The capability of the language model for identifying grammatical Mandarin Chinese text resolves the ambiguity in pronunciation to character mapping, and for the syllable element predictors, which in turn significantly improves the accuracy of the final sentence prediction. While our language model is intended for open and unrestricted Mandarin Chinese language communication, it is worth noting that in specific environments and scenarios, such as in the domestic settings of individuals with disabilities, the language model can be trained on a restricted, predefined corpus to yield more accurate output results.

In this study, the proposed model focuses on decoding neural activity associated with phonetic movements, emphasizing low-level articulatory features such as initials, finals, and tones. To avoid prematurely introducing semantic information, the training corpus consists solely of 407 Mandarin syllables, without explicit semantic content. By decoding these syllabic components, the model captures articulatory representations accurately. However, semantic information is not neglected; it is introduced post-decoding through an *n*-gram model and an LLM. These post-processing steps not only correct potential decoding errors at the syllable level but also ensure grammatical correctness and accurate pronunciation-to-character mappings. This design allows the model to balance low-level phoneme decoding with higher-level syntactic and semantic consistency. Furthermore, the training processes of the AAI and NAR modules are formulated as multi-objective optimization problems, where acoustically inspired components function as regularization terms rather than strict constraints, enabling the model to prioritize phoneme decoding while still retaining the capacity to capture potential semantic cues.

For task design, we used a consistent carrier sentence to minimize variability in the target words caused by different contexts. Embedding target words within this sentence also promotes natural speech flow, reducing the influence of “list intonation”—the unnatural tone, stress, and rate variations seen in isolated word reading [50]. This approach also aids in clear segmentation and annotation, improving preprocessing efficiency. We excluded decoding of neutral tones, as they typically evolve contextually from full-tone counterparts in spoken language [51], although their written form remains unchanged [52]. Future studies may explore neutral tones further in Mandarin Chinese BCI research. To address individual pronunciation differences, we employed participant-specific decoders, training each model exclusively on individual data. This ensures that unique pronunciation patterns are captured, and since all participants read the same materials, these differences do not systematically affect syllable decoding. While minor issues may arise if participants confuse phonetic elements (e.g., “s” and “sh”), our participants exhibited clear and fluent pronunciation, minimizing such effects.

We observed notable variations in the decoding performance when distinguishing between single finals, compound finals, and nasal finals, with the latter 2 showing less favorable results. This may be attributed to the finer timing and position properties of articulatory gestures during a continuous shift of vowels and nasals in compound finals and nasal finals compared to single finals [53]. Thus, we implemented a *k*-means clustering approach, which uses F1 and F2 frequency parameters to classify these nonsingle finals into 11 clusters. These clusters share similar acoustic properties and articulatory patterns, such as “in” and “ing”, grouped based on the nucleus in the structure of finals, as the nucleus bears highest sonority scale [54], for simplifying the decoding process. These can provide a foundation for further investigations into the complexities of decoding compound finals and nasal finals in the context of Mandarin Chinese BCI systems.

In the future, our approach can be adapted for imagined speech decoding, even for participants who cannot speak. The AAI (articulation attribute inference) module, which relies on articulation attributes of Mandarin initials, functions independently of audio and can work without audible speech. While the NAR (neural-acoustic representation) module currently uses formant features from speech recordings, it could be extended by substituting participant-specific recordings with synthetic speech generated via text-to-speech (TTS) systems, provided standard Mandarin pronunciation is used. Additionally, style transfer techniques could align synthetic speech with a participant’s unique acoustic profile, as demonstrated by Metzger et al. [9]. These adaptations offer a promising pathway for imagined speech decoding, including for individuals with aphasia or other speech impairments.

Addressing the challenges of mitigating the need for extensive data acquisition to facilitate cross-day model transfer was beyond the immediate scope of this study. Nonetheless, this endeavor stands as a pivotal requirement in the development of a genuinely pragmatic and impactful system. Furthermore, it is essential to verify the online decoding performance, despite the promising outcomes of our offline analyses. Recent LLMs benefit from extensive training data [55], but combining data across subjects and time points for neurophysiological signals does not yield commensurate enhancements due to inherent cross-participant and cross-day variations. Considering the model adaptation in a lightweight manner to ensure cross-day accuracy and enhancing decoder performance through the use of multi-subject data, the critical endeavor of optimizing the efficient utilization of data from diverse days and individuals remains a key focus for forthcoming research.

## Conclusion

Overall, this study demonstrates a novel approach to offline decoding Mandarin Chinese, a complex logosyllabic language, from brain activity into coherent sentences. By leveraging unique acoustic features of Mandarin syllables and integrating advanced neural network models, we achieved a median character accuracy of 71.00%, with 30.00% of sentences decoded completely accurately. This method highlights the critical role of both cortical and subcortical brain signals and the importance of acoustic-related features in enhancing prediction accuracy. Future research will focus on improving cross-day model transfer, online decoding performance, and optimizing data utilization from multiple subjects and sessions.

## Data Availability

In accordance with the stipulations of clinical ethics protocols, the raw data relevant to this study, including audio data and neurophysiological data, cannot be made publicly available. The electrophysiological data can be obtained upon reasonable request to the corresponding author. To safeguard the anonymity of participants, any information that could identify an individual will not be included as part of the shared data. Any provided data should be kept confidential and should not be shared with others. Source data for figures are provided with the paper.
