# Supplementary material for: Acoustic Inspired Brain-to-Sentence Decoder for Logosyllabic Language
Source: Cyborg Bionic Syst. 2025 Apr 29;6:0257. doi: 10.34133/cbsystems.0257 (PMC12038182; doi:10.34133/cbsystems.0257)
Supplement: Supplementary 1 — Figs. S1 to S6 Tables S1 to S5 [file cbsystems.0257.f1.docx]

SUPPLEMENTARY MATERIALS

**
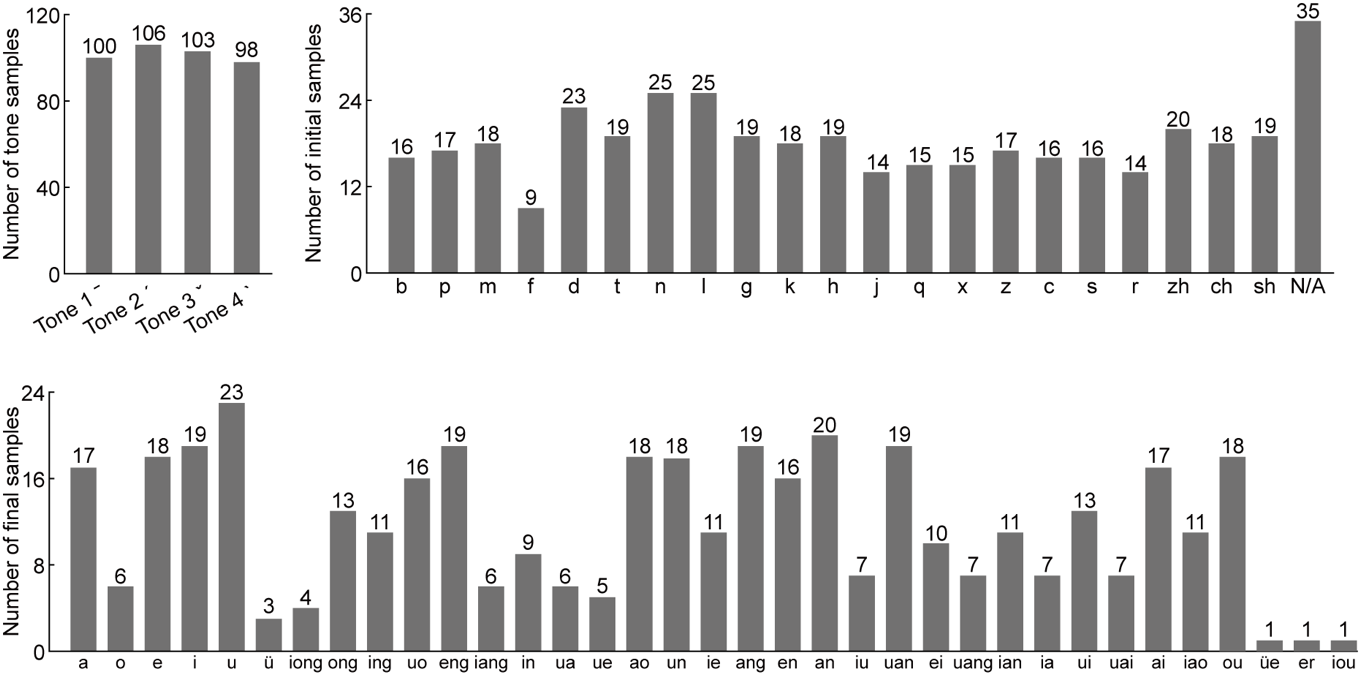
**

Figure S1. Each syllable consists of a tone, a final (rhyme), and at most one initial (with some syllables lacking an initial). The charts display the distribution of the number of initials, tones, and vowels across the 407 syllables (the experimental tasks used for training the three element prediction models).


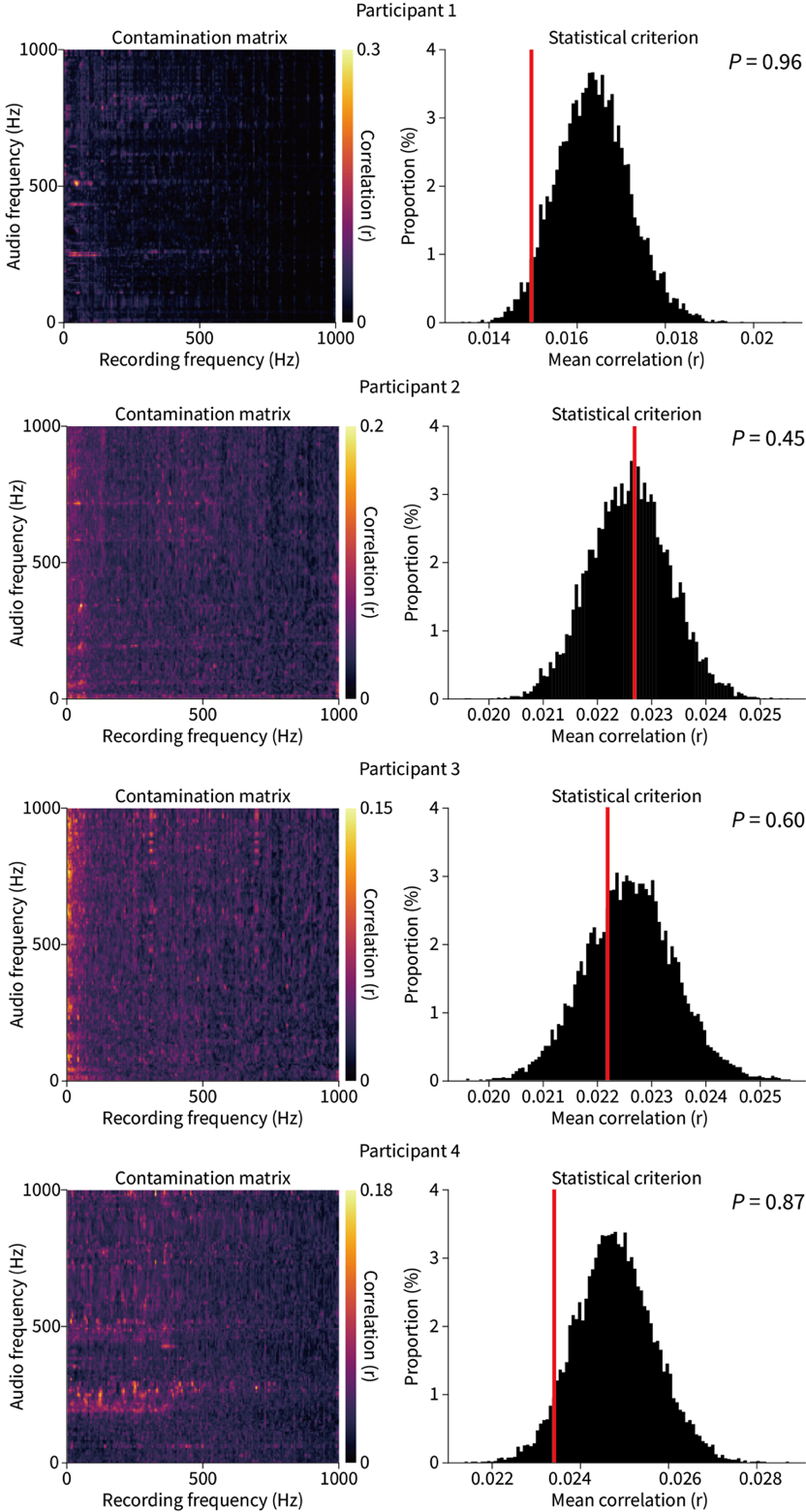


Figure S2. Objective assessment of acoustic contamination in the neural signal for four participants. The heatmap on the left represents the contamination matrix for the neural data of each participant. Brighter colors indicate higher correlation. The graph on the right represents a statistical assessment of acoustic contamination in the neural data for each patient. It compares the average value of the diagonal of the contamination matrix to the distribution of this value in 10,000 randomly shuffled contamination matrices (P-values are displayed within the figures, one-sided).


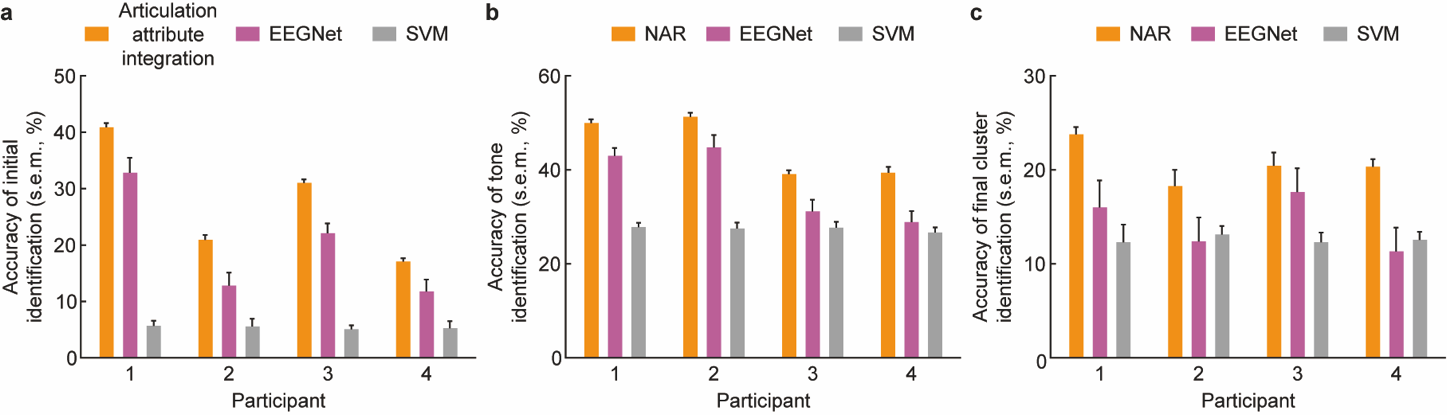


Figure S3. The prediction accuracy (mean ± s.e.m.) for initial (a), tone (b) and final cluster (c) was measured across four participants using a five-fold cross-validation approach.

**
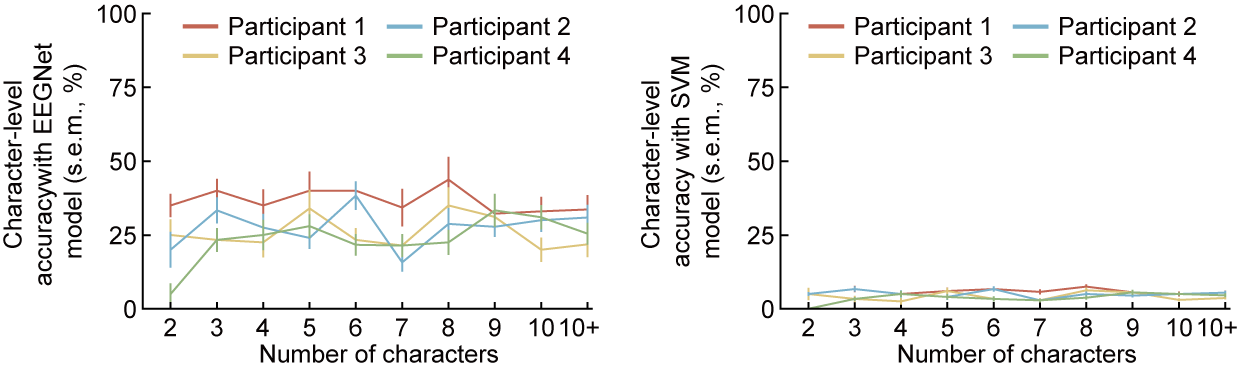
**

**Figure S4.** The character-level accuracy (mean ± s.e.m.) of sentences composed of different lengths based on EEGNet and SVM model.

**
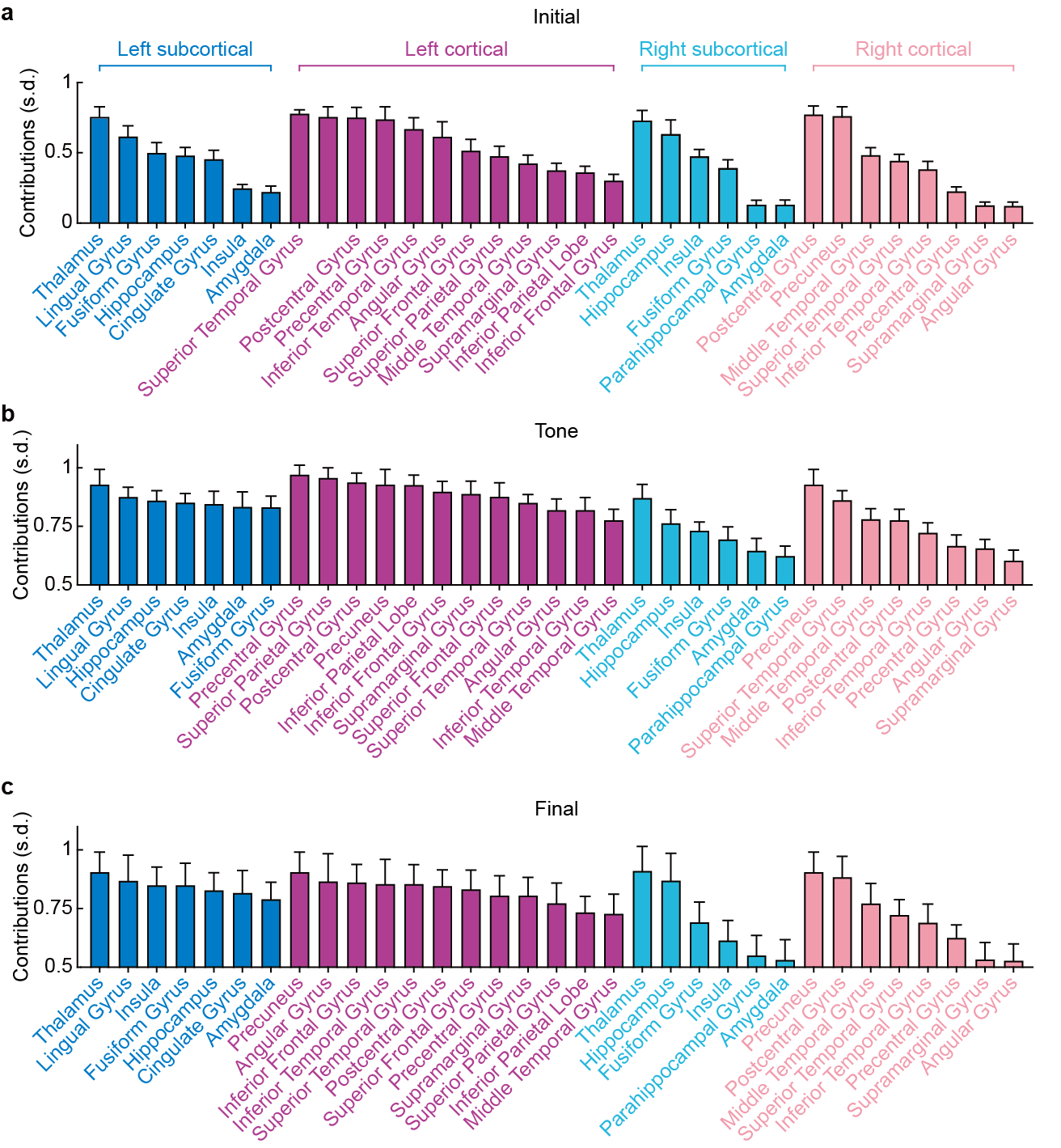
**

**Figure S5.** The contributions of each anatomical area in cortical and subcortical structures of right and left hemispheres of the brain to predicting initials **(a)**, tones **(b)**, and finals **(c)**, as measured by the gradient of the loss function with respect to the input data. The contributions of different participants to the same anatomical area are grouped together.


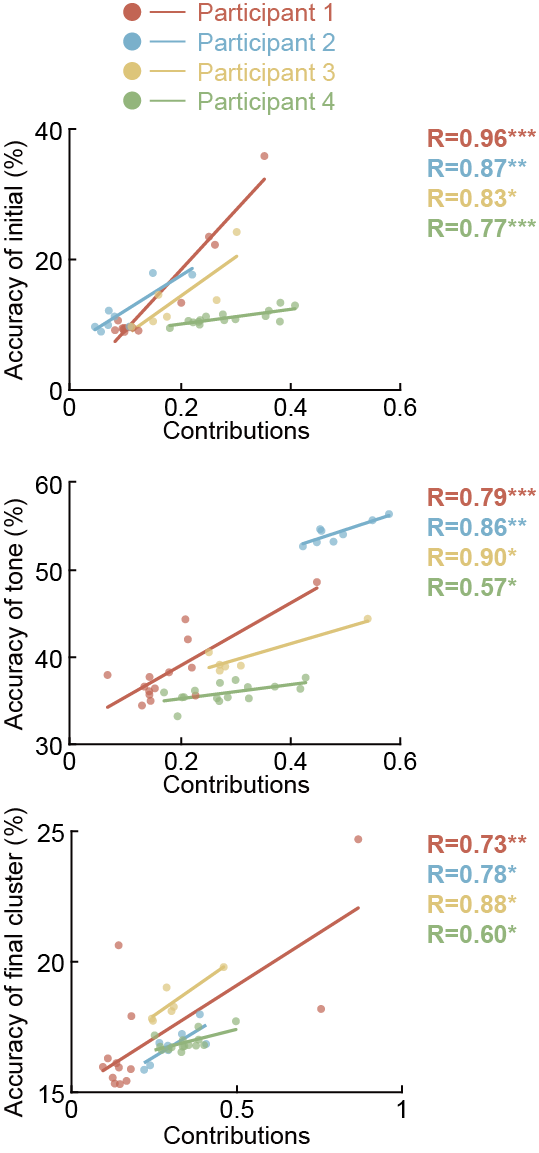


Figure S6. The correlation between prediction accuracies and the corresponding contributions of each anatomical area in three syllable elements prediction models. The Pearson’s correlation coefficient (R) of three syllable elements is calculated separately, with different colors representing different participants (corresponding to Fig. 1). The contributions are measured by the gradient of the loss function with respect to the input data. Each dot represents a brain region's contribution and decoding accuracy for prediction models of initial, tone, and final cluster. **P* < 0.05; ***P* < 0.01; ****P* < 0.0001.

Table S1. Examples of decoded sentences

| Character error rate | Decoded sentences |
| --- | --- |
| 0% | O: 我对自己很有信心 (I have confidence in myself)  D: 我对自己很有信心 |
| ~25% | O: 不喜欢打电话语音 (Don't like voice calls)  D: 不喜欢打电话预约 |
|  | O: 我在自己家里 (I live in my own home)  D: 我住自己家里 |
| ~50% | O: 家庭经济条件不是很可以 (The family financial situation is not very good)  D: 集体经济条件不是还可以 |
|  | O: 一点自我保护意识都没有 (No sense of self-preservation at all)  D: 有点自我保护意识一米五 |
| ~75% | O: 有时也紧张 (Sometimes I also feel nervous)  D: 越说越紧张 |
|  | O: 多多少少也对自己有些影响 (It also has some impact on me to a certain extent)  D: 多多少少有点着急医学影像 |
| >75% | O: 建议尽早到医院接受治疗 (Recommend to seek treatment at the hospital as soon as possible)  D: 建议就知道一样举手之劳 |

All examples are from four participants, which are shown for several character error rate levels. The ‘O’ represents the original sentences and the ‘D’ represents the decoded sentence. The translated English sentences from Mandarin Chinese are provided in parentheses. Any incorrect character in the decoded sentence is marked in red.

Table S2. The articulation features of Mandarin Chinese initials

| Manner of articulation (MOA) | | Plosive | | Affricate | | Fricative | | Nasal | Lateral |
| --- | --- | --- | --- | --- | --- | --- | --- | --- | --- |
| Devoice | | Voiceless | | Voiceless | | Voiceless | Voiced | Voiced | Voiced |
| Aspiration | | Unaspirated | Aspirated | Unaspirated | Aspirated | NA | NA | NA | NA |
| Place of articulation (POA) | Bilabial | b [p] | p [pʰ] |  |  |  |  | m [m] |  |
|  | Labiodental |  |  |  |  | f [f] |  |  |  |
|  | Alveolar | d [t] | t [tʰ] | z [ts] | c [tsʰ] | s [s] |  | n [n] | l [l] |
|  | Retroflex |  |  | zh [ʈʂ] | ch [ʈʂʰ] | sh [ʂ] | r [ʐ] |  |  |
|  | Alveolo-palatal |  |  | j [tɕ] | q [tɕʰ] | x [ɕ] |  |  |  |
|  | Velar | g [k] | k [kʰ] |  |  | h [x] |  |  |  |

Table S3. Detailed architecture specifications for the convolutional neural network utilized in this work.

|  | ConvNet |
| --- | --- |
| Stem | $\boldsymbol{K\times1, 64, stride S}$ |
| Block 1 | $\left[ \begin{matrix} \boldsymbol{7\times1, 128} \\ \boldsymbol{MaxPool, stride 2} \end{matrix} \right]\boldsymbol{\times1}$ |
| Block 2 | $\left[ \begin{matrix} \boldsymbol{7\times1, 128} \\ \boldsymbol{MaxPool, stride 2} \end{matrix} \right]\boldsymbol{\times1}$ |
| Block 3 | $\left[ \begin{matrix} \boldsymbol{7\times1, 256} \\ \boldsymbol{MaxPool, stride 2} \end{matrix} \right]\boldsymbol{\times1}$ |
| Block 4 | $\left[ \boldsymbol{7\times1, 256} \right]\boldsymbol{\times1}$ |

Table S4. Architecture network and training parameters for each participant

| Participant | 1 | 2 | 3 | 4 |
| --- | --- | --- | --- | --- |
| Kernel size | 7 | 7 | 5 | 7 |
| Stride | 4 | 5 | 3 | 5 |

Table S5. Optimizing setup for syllable element prediction.

| Configuration | ConvNet |
| --- | --- |
| Training epochs | 80 |
| Optimizer | AdamW |
| Base learning rate | 0.0003 |
| Weight decay | 0.01 |
| Optimizer momentum | $\boldsymbol{\beta}_{\boldsymbol{1}}\boldsymbol{=0.9}$, $\boldsymbol{\beta}_{\boldsymbol{2}}\boldsymbol{=0.009}$ |
| Batch size | 32 |
| Learning rate schedule | cosine decay |
| Dropout ratio | 0.3 |
| Balancing ratio | $\boldsymbol{\alpha=0.5}$ |
| Degree of freedom | $\boldsymbol{\nu=100}$ |
